# Prolonged Recovery Time from Zoledronic Acid Induced Acute Tubular Necrosis: A Case Report and Review of the Literature

**DOI:** 10.1155/2013/651246

**Published:** 2013-07-29

**Authors:** Frederic Rahbari-Oskoui, Odicie Fielder, Nima Ghasemzadeh, Randolph Hennigar

**Affiliations:** ^1^Renal Division, Department of Medicine, Emory University School of Medicine, Woodruff Memorial Building (WMB) No. 2217, Atlanta, GA 30322, USA; ^2^Department of Medicine, Harbor-UCLA Medical Center, Torrance, CA 90502, USA; ^3^Department of Pathology and Laboratory Medicine, Emory University School of Medicine, Atlanta, GA 30322, USA

## Abstract

Acute tubular necrosis (ATN) due to bisphosphonates has been reported with Zoledronic acid but the time to recovery (if any) has been usually less than 4 months. Possible recovery time from ATN of any cause is usually less than 6 months. In this paper, we present the case of a 59-year-old Caucasian female with metastatic breast cancer who had received 16 monthly injections of Zoledronic acid for treatment of tumor induced hypercalcemia and developed several episodes of mild acute kidney injury which resolved by withholding treatment. Unfortunately, after the sixteenth injection, the patient experienced severe acute kidney injury, with a peak serum creatinine of 8.0 mg/dL. Although urinalysis showed muddy brown casts, because of atypical recovery time and presence of eosinophiluria and subnephrotic range proteinuria, a kidney biopsy was performed. Diagnosis of typical acute tubular necrosis was confirmed without any other concomitant findings. The course was remarkable for an unusually slow recovery of renal function over 15 months without need for renal replacement therapy until the patient expired from her metastatic cancer two years later. We reviewed all the published cases of acute kidney injury due to Zoledronic acid and suggest recommendations for clinicians and researchers.

## 1. Introduction

The use of Zoledronic acid has been reported to cause acute tubular necrosis, but little is known about the particular features of this type of toxic tubular necrosis. 

## 2. Case Report

A 59-year-old Caucasian female with history of type II diabetes mellitus and hypertension was referred to the Emory Renal Clinic for evaluation of increased serum creatinine (SCr). The patient had hypertension for 7 years, type II diabetes mellitus known for 5 years without any documented complications, mild anemia, dyslipidemia, and a baseline SCr of 1.0 mg/dL about 6 months prior to referral. There was no recorded quantification of proteinuria or microalbuminuria since she had been diagnosed with diabetes. Her surgical history was only significant for tonsillectomy. She was single and had never been pregnant. Her medication list included stable doses of Lisinopril, Atenolol, and Pioglitazone. She denied any NSAID use. Her family history was negative for cancers, hypertension, diabetes, or chronic kidney disease. Social history was negative for alcohol, tobacco, or illicit drug abuse. She was a college graduate who worked as a microbiologist. 

The patient had been well until 2 years earlier, when she presented with posttraumatic bruising of her right breast. After resolution of the hematoma a presence of a residual mass led to a breast biopsy which confirmed the diagnosis of infiltrating ductal carcinoma of the right breast. PET scan for staging revealed bone metastases and the cancer was staged as T4N1M1. Estrogen and progesterone receptors were positive and Her-2/neu was negative. Chemotherapy was initiated with 4 cycles of Adriamycin and Cyclophosphamide (AC). Hormonal therapy was then begun with Letrozole (Femara, Novartis). 

Given the bone metastases, monthly infusions of Zoledronic acid (Zometa, Novartis) were begun at the dose of 4 mg IV diluted in 100 cc of normal saline and infused over 30 minutes. Serum Calcium prior to the first dose of Zoledronic acid was 10.3 mg/dL, which was still within the normal range. Zoledronic acid was given for a total of 16 monthly doses of 4 mg IV infused over 15 minutes. Routine labs after the ninth dose revealed a rise in SCr to 2.1 mg/dL and a blood urea nitrogen (BUN) of 18 mg/dL. Zoledronic acid infusions were held for 2 months until SCr returned to baseline. Then treatment was resumed at the same previous dose and continued for 7 months until SCr rose gradually to 1.5 mg/dL. At that point infusions were held again with a return of SCr to 1.1 mg/dL. At that point the patient received 3 more doses and SCr rose to 5.9 mg/dL and BUN to 96 mg/dL. Then the decision was made to permanently discontinue Zoledronic acid. Sodium polystyrene was given and bicarbonate initiated at that visit and the patient was referred to the Nephrology Clinic for evaluation of acute kidney injury. Upon initial evaluation, she felt very well and denied having any pruritus, fatigue, anorexia, nausea/vomiting, decreased urine output, dysuria, polyuria, or dyspnea. On examination, blood pressure was 128/78, with HR of 81. Physical examination was otherwise unremarkable. Urinalysis showed 2+ protein, specific gravity of 1.020, and 1+ leukocyte esterase, with no blood, nitrites, or bacteria. Urine sediment showed numerous coarse granular casts, consistent with acute tubular necrosis, 5–10 WBCs per high power field, and bacteria consistent with a urinary tract infection. Hansel's stain for eosinophiluria was positive for few eosinophiles. Urine culture grew *Klebsiella pneumonia* and she was treated with a 10-day course of Levofloxacin. Over the next 4 weeks, SCr continued to rise to a peak of 8.0 mg/dL. 

Given the absence of rapid resolution of acute renal failure and onset of subnephrotic range proteinuria (spot urine protein to creatinine ratio of 1.46 g/gram of SCr), a workup for proteinuria was initiated. The exact origin of proteinuria (glomerular versus tubular) was not determined. Serum protein electrophoresis showed an elevated alpha-globulin and slightly elevated alpha-antitrypsin band consistent with acute phase response. Complement levels, ANCA, anti-DNA, and anti-nuclear antibodies were normal. Hepatitis C antibody was negative and she was immune to Hepatitis B. 

Renal biopsy was performed to rule out the possibility of any concomitant disease—more specifically, acute interstitial nephritis, given the presence of mild eosinophiluria. 

## 3. Pathology of the Renal Biopsy

**Figure 1:**
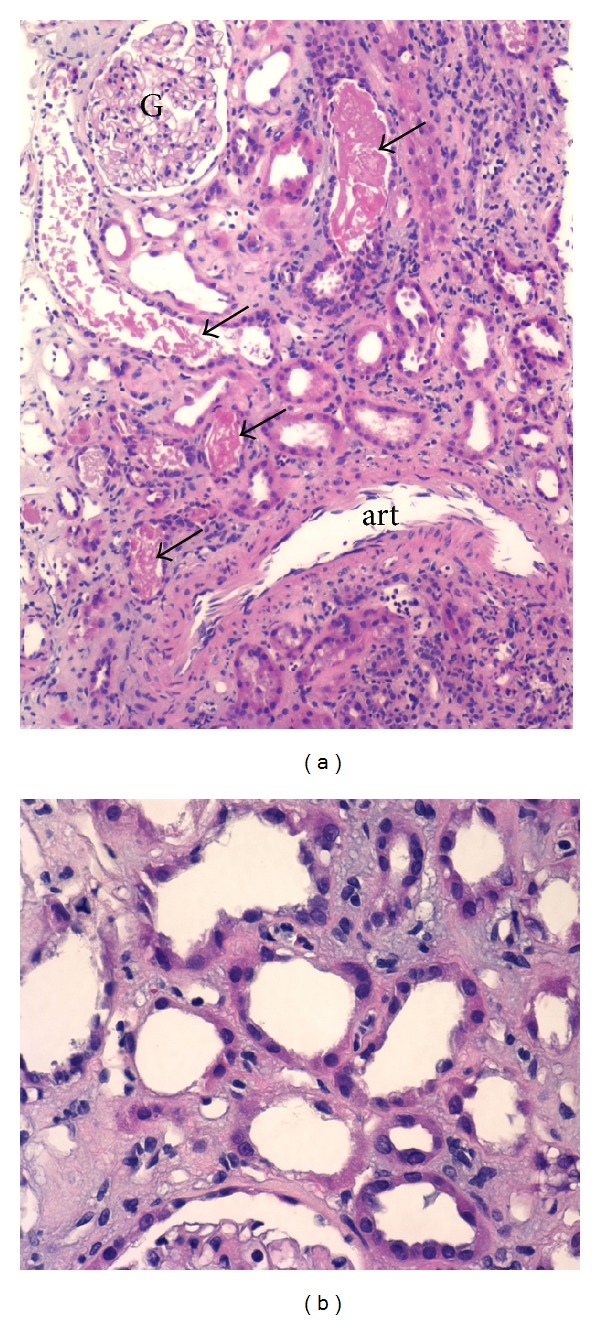
Light microscopy renal biopsy results (H&E stain). Renal biopsy showing changes of ATN. (a) Proximal tubules are mildly dilated and simplified in appearance. Scattered tubules contain denuded and necrotic epithelial cells (arrows). Patchy very mild interstitial inflammation is seen in the right upper-hand corner. Glomeruli (G) exhibit only mild mesangial prominence; art: distal artery (H&E stain, original magnification = 100x). (b) Higher magnification reveals attenuation of proximal tubular epithelium with loss or disruption of brush border. The interstitium exhibits edema superimposed upon mild fibrosis (H&E stain, original magnification = 400x).

The biopsy specimen was submitted for conventional light, immunofluorescence, and electron microscopic studies. Glomerular findings were nonspecific and limited primarily to mild mesangial prominence. Glomerular hypercellularity was nominal with no evidence for podocyte injury or an immune complex-mediated process. Only about 5% of glomeruli were sclerosed. The tubulointerstitium exhibited signs of acute tubular necrosis (ATN) (Figure 1). Proximal tubules were reactive in appearance and mildly dilated. Numerous proximal tubules showed attenuation (simplification) of the epithelium with extensive loss or disruption of brush border. Scattered tubules contained denuded and necrotic epithelial cells, as well as occasional hyaline casts. About 10–20% of tubules were in varying stages of atrophy. The interstitium showed diffuse but mild fibrotic changes. Patchy mild chronic interstitial inflammation was present and consisted mainly of reactive lymphocytes. Plasma cells and eosinophils were not a prominent feature of the inflammatory infiltrate. Electron microscopy was essentially unremarkable, not showing any abnormalities in mesangial compartment, foot processes, or basement membrane. There were only mild endothelial cell fenestration and some increased wrinkling of the peripheral capillary basement membranes. No deposits were found in any compartments. Immunofluorescence studies showed 2+ positive staining to Kappa and Lambda heavy chains otherwise unremarkable (specifically no glomerular staining at all). The overall impression was acute tubular necrosis with some evidence of chronic tubule-interstitial and minimal glomerular scarring.

## 4. Course of the Disease

**Figure 2:**
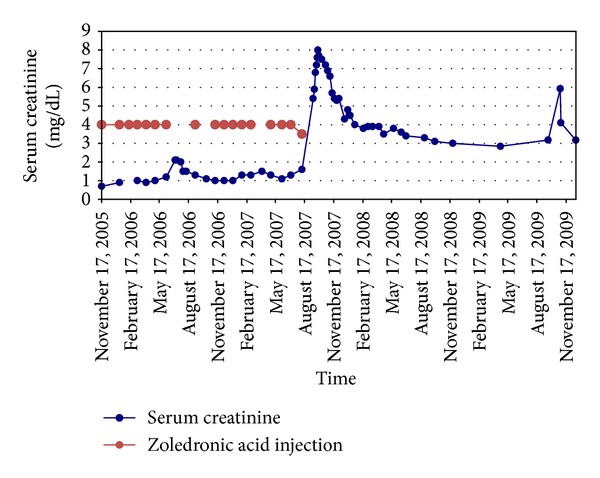
Evolution of serum creatinine in relation to injections of Zoledronic acid.

Zoledronic acid was permanently discontinued and over the next several months SCr started trending down. She was doing very well on Aromasin and Tamoxifen for about 26 months with a stable SCr around 3.0 mg/dL. Unfortunately, she developed ascites and upper GI bleed and the workup showed extensive metastatic spread of her breast cancer to the liver and bones with peritoneal carcinomatosis. She had acute kidney injury due to prerenal azotemia. Because of her grim prognosis hospice care was deemed to be the best option for her care and she passed away 28 months after the last dose of Zoledronic acid without ever needing renal replacement therapy. Interestingly, and despite her extensive bone metastases, she never developed hypercalcemia.  Figure 2 summarizes the evolution of SCr levels before and after injection of Zoledronic acid from 2005 to 2009, and Table 1 shows the other biological measurements. 

## 5. Discussion

We are presenting a case of acute kidney injury secondary to a new member of the bisphosphonate class of medications, Zoledronic acid, with a very unusual recovery course. 

Intravenous Zoledronic acid has been approved by the US Food and Drug Administration in metastatic bone disease, malignancy related hypercalcemia, multiple myeloma, and Paget's disease of the bone. The use of lower dose infusions of Zoledronic acid has also been suggested in osteoporosis in order to improve therapeutic compliance [5, 6]. 

Mechanistically, bisphosphonates are known to be potent calcium chelating agents. Once internalized by osteoclasts, bisphosphonates inhibit a critical step in the mevalonate pathway, which is involved in lipid alteration of small molecule GTPases [7, 8]. This mechanism disrupts the function within osteoclasts, leading to cellular apoptosis, but has also been implicated in the death of tubular cells in acute tubular necrosis [9]. Even though the oral formulations of bisphosphonates seem to spare the kidneys, the intravenous use of them has been associated with several types of renal injury including collapsing focal and segmental glomerulosclerosis, acute tubular necrosis, and also “minimal change disease” [10]. Zoledronic acid is a newer generation of bisphosphonates which is favored for its increased ability to inhibit bone resorption [11] and has been demonstrated to have greater bone resorptive capacity over older agents, including Pamidronic acid [9, 12]. 

Phase III trials have elucidated the renal toxicity of Zoledronic acid. Calcium chelation induces formation of an insoluble calcium phosphate product, which then precipitates in the proximal tubule [13]. Zometa is eliminated, unmetabolized, by the kidney via glomerular filtration and tubular excretion [14]. There is a positive correlation between both the injected dose and the duration of infusion and occurrence of acute kidney injury. With the optimal intravenous infusion regimen of 4 mg over 15 minutes, acute kidney injury has been reported in less than 10% of patients [7–15]. 

Ibandronate, a newer member of the nitrogen-containing bisphosphonate class, has been suggested to have a more favorable toxicity profile than Zoledronic acid in some trials [16, 17] and, possibly, be associated with minimal renal effects [18, 19]. 

Few particular considerations are of clinical importance in this case. First, despite receiving Zometa at the recommended low dose of 4 mg/month with slow infusion rate, this patient developed several episodes of acute kidney injury which resolved after holding the infusions for one to two months. Ultimately, severe permanent kidney injury occurred. Of note, this patient's SCr was already doubled (1.6 mg/dL compared to baseline of 0.8 mg/dL) before even receiving the final dose of treatment, which retrospectively should have raised concerns. This mild impairment in renal function, in combination with a possible cumulative dose effect (after 16 doses of 4 mg), was potential risk factor for occurrence of acute renal injury. Therefore, any degree of renal failure should prompt consideration of holding or discontinuing therapy. In addition, it is unclear what level of preexisting glomerular disease or susceptibility may have been present based on the patient's known comorbidities (diabetes and hypertension) or malignancy. In two series reports by Chang et al. and others [20, 21], associated factors contributing to renal failure were advanced cancer, receipt of chemotherapy, multiple myeloma, concomitant use of nephrotoxins, chronic renal failure, diabetes, and hypertension. Hypercalcemia, although not present at baseline in our patient, is a known complication of metastatic bone disease and may increase renal susceptibility to hypoperfusion via vasoconstriction of the afferent arteriole. In addition, hypercalcemia-induced osmotic diuresis can promote a prerenal condition, and nephrocalcinosis is another mechanism of renal toxicity in that clinical setting. 

Second, to our knowledge, the unusually slow pace of recovery from ATN, which took more than 14 months without reaching a plateau, has not been reported in any other etiology of ATN. Whether this is due to the unusually long tissue half life of Zoledronic acid is unclear, but SCr levels continued to rise even after discontinuation of therapy. In a French registry, the longest time to resolution was reported as 9 months in one patient, with considerable variation in range, with the lower end being 2 days [21]. An individual report by Koike et al. showed recovery of the renal function after 7 months of dialysis-dependant renal failure due to ATN caused by Zoledronic acid [3]. 

Third, without having definitive indications for renal replacement therapy, initiation of hemodialysis should be kept as a last option and spontaneous recovery of renal function by complete discontinuation of the treatment and medical management of fluid and electrolyte abnormalities is possible. 

Finally, it is also important to note that eosinophiluria and mild proteinuria can be present in case of Zoledronic acid induced ATN. 

Our review of the literature on reported cases of acute renal failure due to Zoledronic acid (see Table 2) reveals that most cases have been reported in a context of multiple myeloma, but few cases have also been reported in Paget's disease, prostate cancer, and breast cancer. The fact that multiple myeloma presents more commonly with overt hypercalcemia may be an explanation of the observed difference. 

Furthermore, the same type of nephrotoxicity has been reported in similar populations after substitution of long term Pamidronic acid therapy by Zoledronic acid [2, 22]. The mean elevation of serum creatinine was 1.5-fold of the baseline level in the case series published by Diel et al. [22]. 

The possibility of a better alternative by using nitrogen-containing bisphosphonates was investigated in a retrospective fashion by Ingo et al. They showed a 1.5-fold increase in relative risk of acute kidney injury with the use of Zometa compared to ibandronate [22]. The study suffered from a significant difference in the two arms of the study in duration of observation of patients and concomitant use of drugs with nephrotoxic moieties. Additionally, a single case report where ibandronate was used as a substitution therapy to Zoledronic acid after occurrence of renal injury resulted in full recovery of renal injury [4]. 

## 6. Conclusion

Based on these observations, we conclude that presence of previous reversible episodes of mild acute kidney injury should be considered as a risk factor for occurrence of severe kidney injury. Furthermore, in absence of clear indication, or a less toxic alternative, the use of Zoledronic acid should be avoided with any level of acute kidney injury or advanced chronic kidney disease. Ibandronate may represent a safer agent in those situations. The recovery time from acute tubular necrosis may be extremely long (15 months) due to extremely long half life of the agent. Presence of impressively high levels of serum creatinine does not warrant the initiation of dialysis in absence of other indications for acute dialysis. Randomized clinical trials comparing different bisphosphonates are needed to establish potential superiority of a specific agent; special attention should be paid to the possibility of a toxic cumulative dose of Zoledronic acid and to determine appropriate interventions for patients with renal impairment. 

## Figures and Tables

**Table 1:** Laboratory data values.

Variable	Baseline	Nephrology referral	7 weeks after last dose	14 months after last dose
Creatinine (mg/dL)	0.8	5.9	8.0	3.1
BUN (mg/dL)	18	96	55	51
Sodium (mEq/L)	138	135	132	139
Potassium (mEq/L)	4.5	6.1	3.6	3.3
Chloride (mEq/L)	100	108	95	101
Bicarbonate (mEq)	29	15	22	24
Calcium (mg/dL)	10.3	10.3	9.5	9.5
Albumin (g/dL)	4.5	4.1	3.6	3.6
Hemoglobin (g/dL)	12.9	10.5	9.9	10.6
U.P/C (g/g of creat.)	Unknown	91/99	111/76	63/89

BUN: blood urea nitrogen.

U.P/C: random urinary protein to creatinine ratio.

**Table 2:** Profile of reported cases of ARF associated with Zoledronic acid.

Author	Case	Underlying disease	Baseline creatinine (mg/dL)	Bisphosphonate use prior to Zometa	Dose of Zometa (mg)	Recovery time (months)	Dialysis requirement (yes/no)	Serum creatinine peak/end (mg/dL)
Ramazzina et al. [1]	1	MM	U/K	Pamidronate × 4 doses	4 mg	9	Yes	ESRD
Markowitz et al. [2]	2	MM	1.5	Pamidronate × 13 doses	4 mg	4	No	4.0/2.4
Markowitz et al. [2]	3	Paget's	1.5	Pamidronate × 10 doses	4 mg	4	No	3.8/2.6
Markowitz et al. [2]	4	MM	1.3	Pamidronate × 45 doses	4 mg	9	No	2.5/2.3
Markowitz et al. [2]	5	MM	1.4	Pamidronate × 22 doses	4 mg	4	No	2.6/1.6
Markowitz et al. [2]	6	MM	1.5	Pamidronate × 2 doses	4 mg	3	No	5.5/3.0
Markowitz et al. [2]	7	Prostate Cancer	1.0	Pamidronate × 9 doses	4 mg	4	No	2.0/1.7
Koike et al. [3]	8	MM	U/K	None	4 mg	7	Yes	7.3/UK
Joensuu [4]	9	Prostate Cancer	0.7	None	4 mg	5	No	1.2/0.7
Our patient	10	Breast Cancer	0.8	None	4 mg	>14	No	8.0/3.0

MM: multiple myeloma.

ARF: acute renal failure.

## References

[1] Ramazzina C, Aschmann YZ, Kummer O, Bravo AER, Bodmer M (2007). Zoledronate-associated end stage renal failure and hypocalcaemia. *Praxis*.

[2] Markowitz GS, Fine PL, Stack JI (2003). Toxic acute tubular necrosis following treatment with zoledronate (Zometa). *Kidney International*.

[3] Koike K, Fukami K, Morishige S (2009). A case of acute kidney injury related to intravenous zoledronic acid in a patient with multiple myeloma. *Japanese Journal of Nephrology*.

[4] Joensuu TK (2008). Renal toxicity following zoledronic acid reversed with ibandronate in a prostate cancer patient with bone metastases. *Urologia Internationalis*.

[5] Conte P, Guarneri V (2004). Safety of intravenous and oral bisphosphonates and compliance with dosing regimens. *Oncologist*.

[6] Guarneri V, Donati S, Nicolini M, Giovannelli S, D’Amico R, Conte PF (2005). Renal safety and efficacy of i.v. bisphosphonates in patients with skeletal metastases treated for up to 10 years. *Oncologist*.

[7] Banerjee D, Asif A, Striker L, Preston RA, Bourgoignie JJ, Roth D (2003). Short-term, high-dose pamidronate-induced acute tubular necrosis: the postulated mechanisms of bisphosphonate nephrotoxicity. *American Journal of Kidney Diseases*.

[8] Lin JH (1996). Bisphosphonates: a review of their pharmacokinetic properties. *Bone*.

[9] Lühe A, Künkele K, Haiker M (2008). Preclinical evidence for nitrogen-containing bisphosphonate inhibition of farnesyl diphosphate (FPP) synthase in the kidney: implications for renal safety. *Toxicology in Vitro*.

[10] Markowitz GS, Appel GB, Fine PL (2001). Collapsing focal segmental glomerulosclerosis following treatment with high-dose pamidronate. *Journal of the American Society of Nephrology*.

[11] Green JR, Seltenmeyer Y, Jaeggi KA, Widler L (1997). Renal tolerability profile of novel, potent bisphosphonates in two short-term rat models. *Pharmacology and Toxicology*.

[12] Body J, Pfister T, Bauss F (2005). Preclinical perspectives on bisphosphonate renal safety. *Oncologist*.

[13] Zojer N, Keck AV, Pecherstorfer M (1999). Comparative tolerability of drug therapies for hypercalcaemia of malignancy. *Drug Safety*.

[14] (2005). *Zometa (Package Insert)*.

[15] Rosen LS, Gordon D, Kaminski M (2003). Long-term efficacy and safety of zoledronic acid compared with pamidronate disodium in the treatment of skeletal complications in patients with advanced multiple myeloma or breast carcinoma: a randomized, double-blind, multicenter, comparative trial. *Cancer*.

[16] Pfister T, Atzpodien E, Bauss F (2003). The renal effects of minimally nephrotoxic doses of ibandronate and zoledronate following single and intermittent intravenous administration in rats. *Toxicology*.

[17] Bell R (2005). Efficacy of ibandronate in metastatic bone disease: review of clinical data. *Oncologist*.

[18] Heidenreich A, Ohlmann C, Olbert P, Hegele A (2003). High-dose ibandronate is effective and well tolerated in the treatment of pain and hypercalcemia due to metastatic urologic cancer. *European Journal of Cancer*.

[19] Ohlmann C, Heidenreich A (2003). Efficacy of ibandronate in the management of painful osseous metastases due to hormone refractory prostate cancer. *Supportive Care Cancer*.

[20] Chang JT, Green L, Beitz J, Tarassoff P, Hei Y, Maladorno D (2003). Renal failure with the use of zoledronic acid. *The New England Journal of Medicine*.

[21] Munier A, Gras V, Andrejak M (2005). Zoledronic acid and renal toxicity: data from French adverse effect reporting database. *Annals of Pharmacotherapy*.

[22] Diel IJ, Weide R, Köppler H (2009). Risk of renal impairment after treatment with ibandronate versus zoledronic acid: a retrospective medical records review. *Supportive Care in Cancer*.

